# Observations on chromosome-specific sequencing for the construction of cross-species chromosome homology maps and its resolution of human:alpaca homology

**DOI:** 10.1186/s13039-022-00622-0

**Published:** 2022-10-07

**Authors:** Malcolm A. Ferguson-Smith, Jorge C. Pereira, Ana Borges, Fumio Kasai

**Affiliations:** 1 Resource Centre for Comparative Genomics, Department of Veterinary Medicine, University of Cambridge, Madingley Road, Cambridge, CB3 0ES UK; 2 Animal and Veterinary Research Centre (CECAV), AL 4AnimalS, UTAD, Vila Real, Portugal; 3 Cell Engineering Division, BioResource Research Center, RIKEN Cell Bank, Tsukuba, Japan

**Keywords:** Chromosome mapping, Comparative genomics, Chromosome-specific sequencing, Alpaca homology

## Abstract

**Background:**

The history of comparative chromosome mapping is briefly reviewed, with discussion about the problem that occurs in chromosome painting when size heteromorphisms between homologues cause contamination in chromosomes sorted by flow cytometry that are used in the preparation of chromosome-specific DNA probes.

**Main body:**

As an example, we show in the alpaca (*Vicagna pacos*) that sequencing of contaminated chromosome sorts can reveal chromosome homologies from alignment with human and mouse genome reference sequences. The procedure identifies syntenic blocks of DNA separated in the human karyotype that are associated in the closely related alpaca and dromedary (*Camelus dromedarius*) karyotypes. This example provides proof of principal for the validity of the method for comparative chromosome mapping.

**Conclusion:**

It is suggested that the approach presented here may have application in the construction of comparative chromosome maps between distantly related taxa, such as monotremes and mammals.

## Background

In the early days of human gene mapping, it was noted that certain human genetic linkage groups could be found intact in mouse and later in other species [1]. These observations led to an interest in the construction of comparative chromosome maps and, notably, the introduction of what became known as the Oxford grid [1]. The latter gives a useful pictorial summary of the areas of homology between the chromosomes of two selected species. The homologies were identified first by mapping biochemical and DNA markers to chromosomes in man/mouse hybrid cell cultures, which contained only specific human chromosomes. Polymorphisms allowed distinction between the markers of the two species. This technique was followed later by in situ localization of genes to metaphase chromosomes by hybridization of radioactive or fluorescence labelled probes [2]. In the 1990s, the development of plasmid and other chromosome-specific DNA libraries produced by chromosome microdissection enabled cytogeneticists to “paint” specific chromosomes with fluorescent DNA fragments revealing regions of homology when the paints were applied between species [3–5].

Chromosome painting produced unexpectedly large areas of homology, sometimes involving whole chromosomes or chromosome arms between closely related species. These regions are usually referred to as syntenic blocks. In more distantly related species, the blocks are divided into smaller regions that are rearranged in different combinations. In fact, the characteristic karyotype of each species in terms of chromosome number and morphology can be explained largely by rearrangements during evolution in the number, order, and pattern of syntenic associations in each karyotype [6]. We suggest that this may be an important attribute leading to speciation. In many examples of cross-species painting, repetitive DNA fragments derived from ancestral telomeres or centromeres can be detected between syntenic blocks indicating chromosome fusions during evolution between homologous regions on non-homologous chromosomes [7]. This was first shown in the Indian muntjac in which the 2n = 6/7 karyotype revealed fusions homologous to Chinese muntjac chromosomes [7].

The DNA amplification of chromosome-specific libraries for the preparation of chromosome paints was replaced in 1992 by chromosomes sorted in fluid suspension using a dual laser fluorescence-activated cytometer and the sorts amplified using degenerate oligonucleotide primers [8]. The chromosomes are sorted according to length and base-pair ratio and presented as a flow karyotype. The precise separation of specific chromosome pairs into distinct peaks in the flow karyotype allowed the homologues of heteromorphic chromosomes (such as the human acrocentrics) to be distinguished and the presence of chromosome aberrations to be identified. The sorted material was first used for diagnostic cytogenetics [9] and was then applied to comparative mapping projects [5]. Chromosome sorting also allows the measurement of individual chromosome size in any species and thus estimates of genome size from summation of chromosome sizes; the method requires the joint sorting of mixed chromosome preparations from test and control species in which some chromosome sizes are known in the latter. The procedure has been published in studies used to estimate genome sizes in examples of mammalian [10], marsupial [11], avian and reptilian [12, 13] species. Our methods for chromosome sorting, DNA amplification and in situ hybridization are fully described in these reports [12, 13] and are not repeated here.

Patterns of association of syntenic blocks have been important in the determination of the closeness of relationships between taxa, and the path of their evolution from a common ancestor [6]. Thus, the course of mammalian karyotype evolution has been studied in representatives of all mammalian orders [6] and similar studies have demonstrated karyotype evolution within birds [14–16], reptiles [17], fishes [18, 19] and between reptiles and birds [20]. Avian karyotype evolution has been extended even to the construction of the presumptive ancestral karyotype of extinct dinosaurs [21]. If visual proof were needed for Darwin’s theory of the Origin of Species from a common progenitor, comparative chromosome mapping clearly provides it.

In most instances, comparative chromosome painting is a relatively simple procedure. While it works well within mammals, marsupials, monotremes and reptiles, it is not effective between these major phyla due to DNA divergence during evolution. The marsupial X chromosome is an interesting exception as, when painted by the human X probe, its long arm homology is revealed [22]. However, the level of DNA conservation is sufficient between birds and reptiles to permit useful comparative painting [23]. In some mammalian species, such as the alpaca (*Vicugna pacos,* VPA*,* 2n = 74) the procedure presents difficulties due to the presence of variable amounts of repetitive DNA in the homologues of multiple chromosome pairs which causes difficulty in identifying pairs of chromosomes in the karyotype. In this difficult situation, we propose that sequencing of chromosome sorts can be used to make comparative maps, provided that these sequences can be aligned with a reference sequence of the genome of the species with which alpaca or other test species is being compared. Draft genome sequences of an increasing number of species are now available in accessible databases and these make this strategy possible.

As a proof of principal, we describe briefly here our preliminary unpublished studies that demonstrate the value of chromosome-specific sequencing in producing comparative chromosome maps showing regions of alpaca homology in human and mouse chromosomes. The method is applicable to the production of such maps in all situations where chromosome painting is not possible. Indeed, we hope to show that the accuracy of the method for identifying cross-species homology may be superior to chromosome painting and that it is likely to replace the latter in the future.

## The alpaca model

**Fig. 1:**
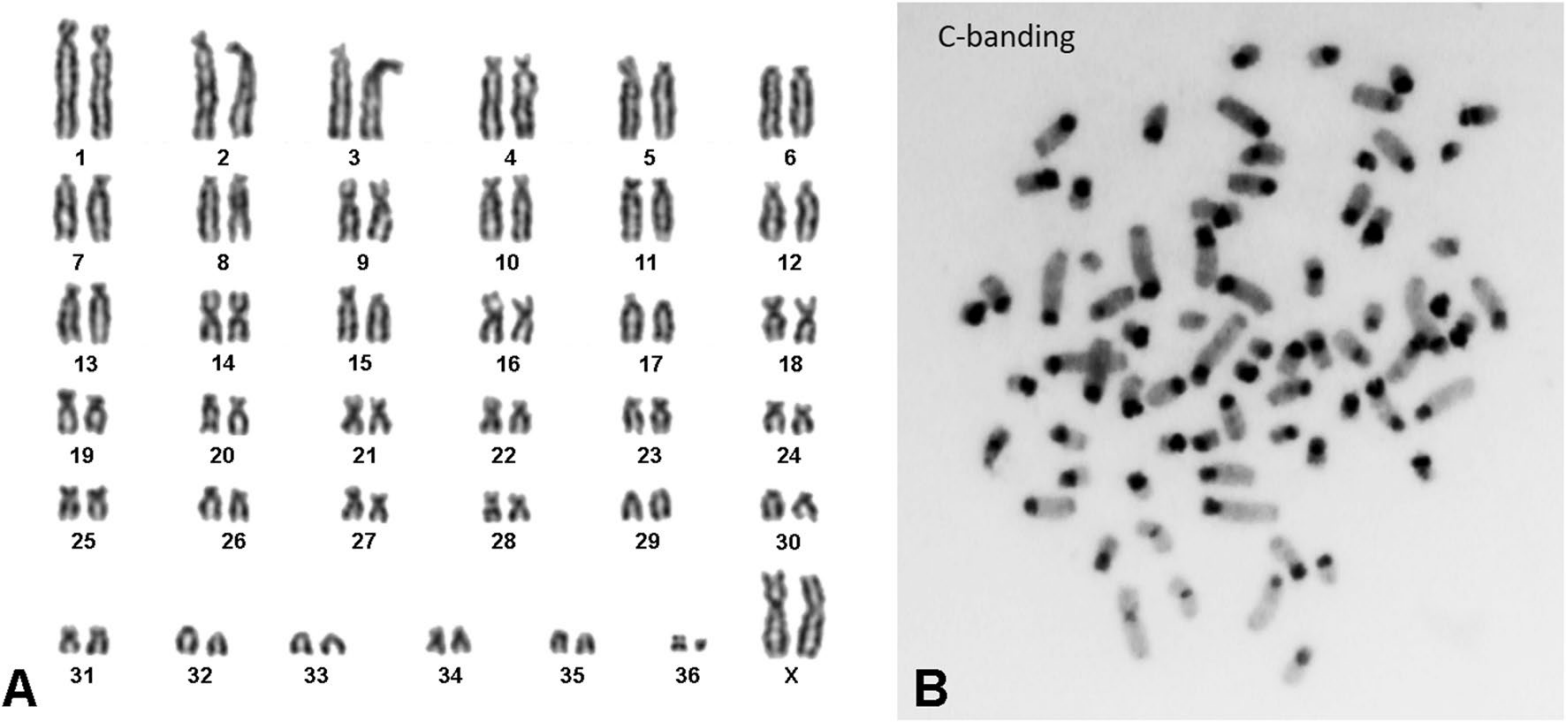
(**A**) The karyotype of female alpaca (modified from [24]); autosomal centromeric heteromorphisms are evident (note presence of minute chromosome 36, possibly a normal variant). (**B**) C-banded metaphase demonstrating chromosomes with extensive, variable centromeric heterochromatin

**Fig. 2:**
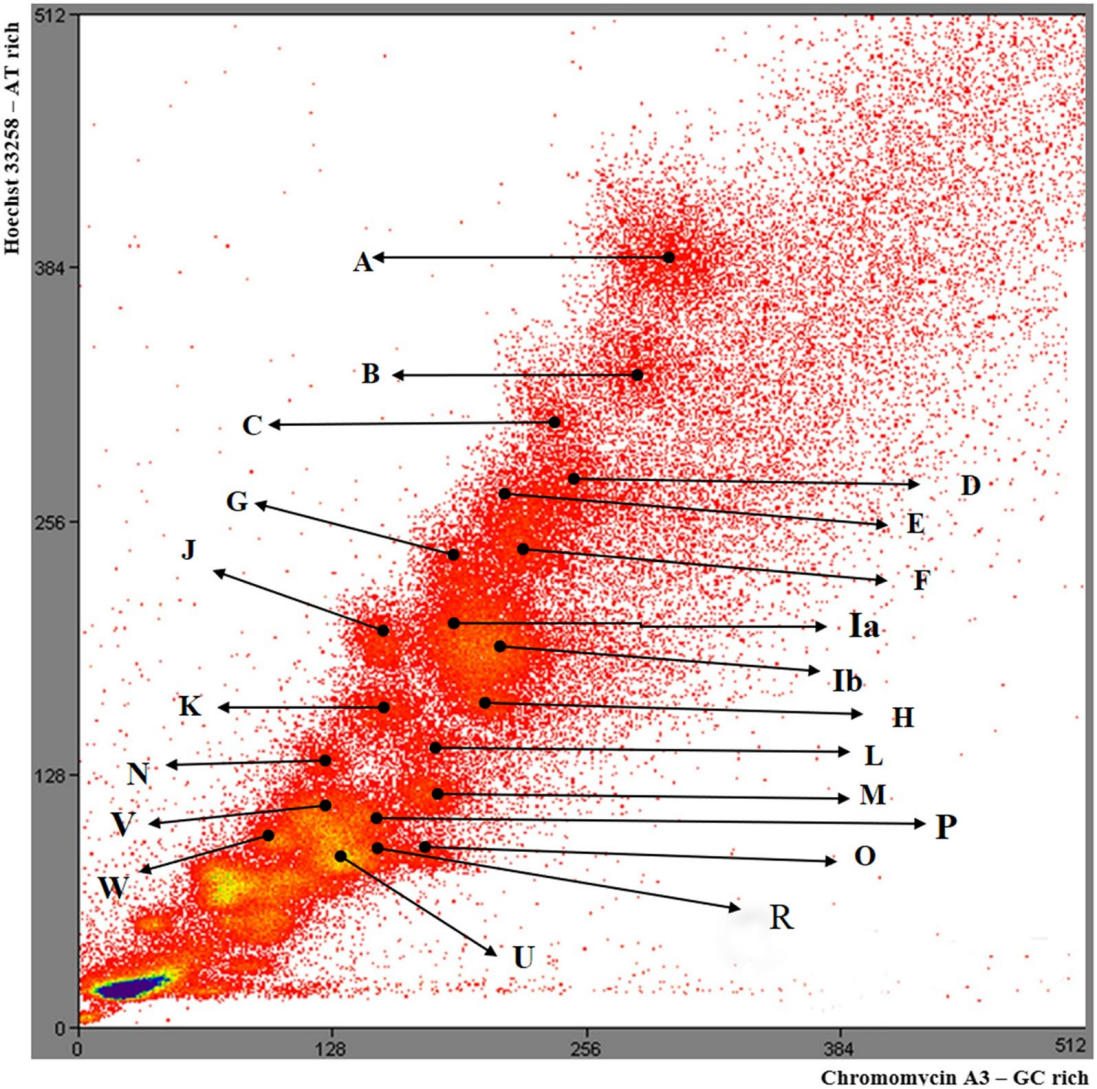
Flow karyotype of alpaca. Peaks represent chromosomes sorted by size. They are labelled alphabetically as several contain chromosomes of more than one pair due to variable amounts of heterochromatin in homologues, causing size differences. Note that there is no peak equivalent to CDR peak 4 in the dromedary (see Fig. 4) as, due to size difference, the equivalent chromosome is present in alpaca VGA peak D

**Fig. 3:**
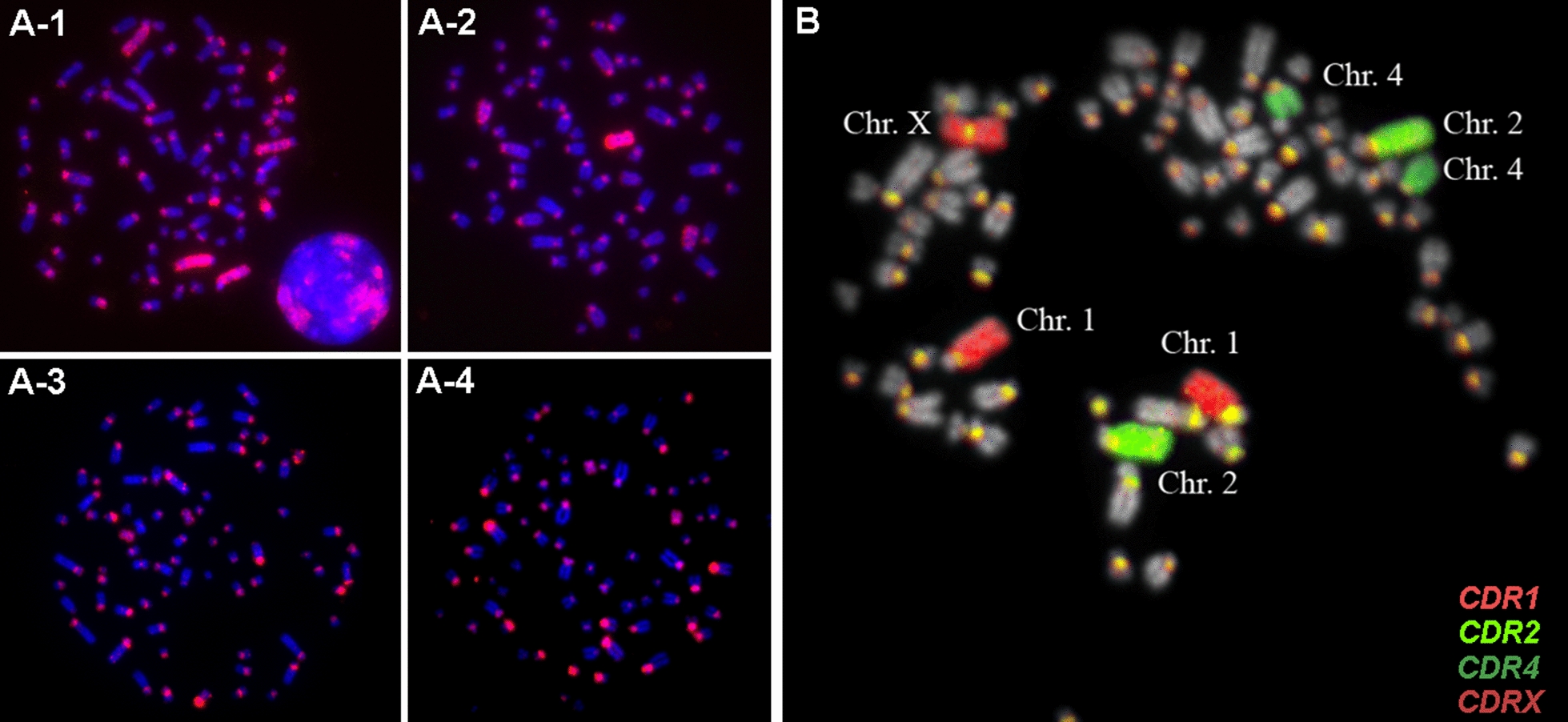
(**A**) Alpaca metaphases hybridized in situ with alpaca paint probes made from flow karyotype peaks A (A-1), C (A-2), M (A-3), and N (A-4). Note signals on multiple chromosomes in each example. (**B**) Alpaca metaphase hybridized in situ with dromedary paint probes (CDR) 1, 2, 4 and X

**Fig. 4:**
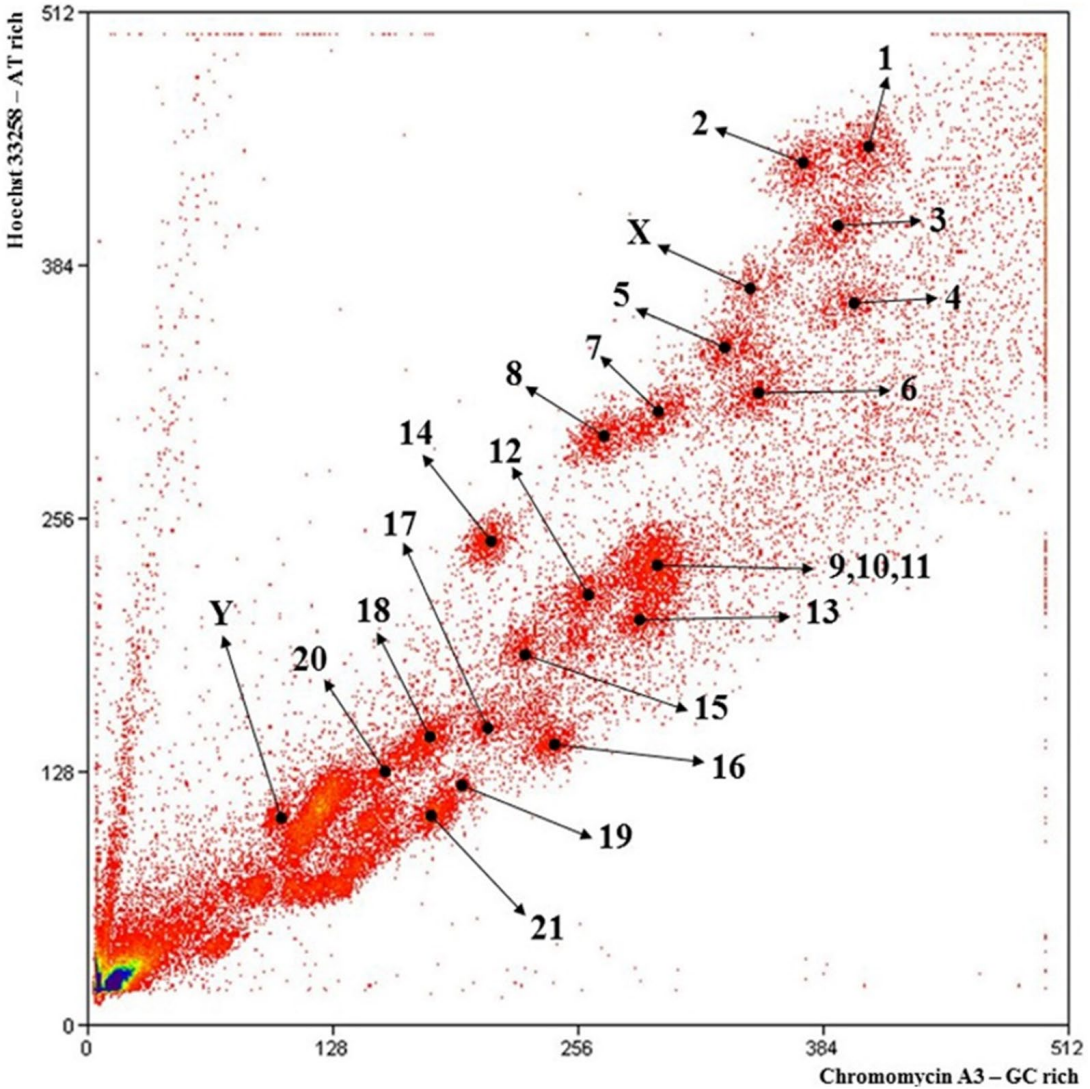
Flow karyotype of dromedary. Peaks are more precise compared to alpaca and are numbered by the chromosome pair present in each peak. Note CDR peak 4, which corresponds to VPA 6 in peak D of the alpaca flow karyotype (Fig. 2)

During the production of alpaca chromosome-specific paints for comparative studies with other camelids, we noted the presence of substantial regions of centromeric DNA repeats throughout metaphase chromosomes, confirmed by C-banding (Fig. 1). As homologous chromosomes differed in size due to differing amounts of these repeats, each peak in the flow karyotype contains samples of more than one chromosome pair and, in some instances, two peaks contain different homologues of the same pair. This leads to diffuse peaks in the alpaca flow karyotype (Fig. 2) and some problems in pairing the chromosomes into a standard karyotype (Fig. 1). DNA probes made from all the sorted alpaca chromosome peaks paint multiple chromosomes (Fig. 3A). In contrast, the flow karyotype of the closely related dromedary (*Camelus dromedarius,* CDR, 2n = 74) shows precise peaks corresponding to single types of chromosome and small groups of chromosomes of the same size (Fig. 4). The dromedary DNA chromosome-specific probes each paint single pairs of alpaca chromosomes (Fig. 3B), indicating a high level of non-repetitive DNA conservation between the two species and the absence of interchromosomal rearrangements.

**Fig. 5:**
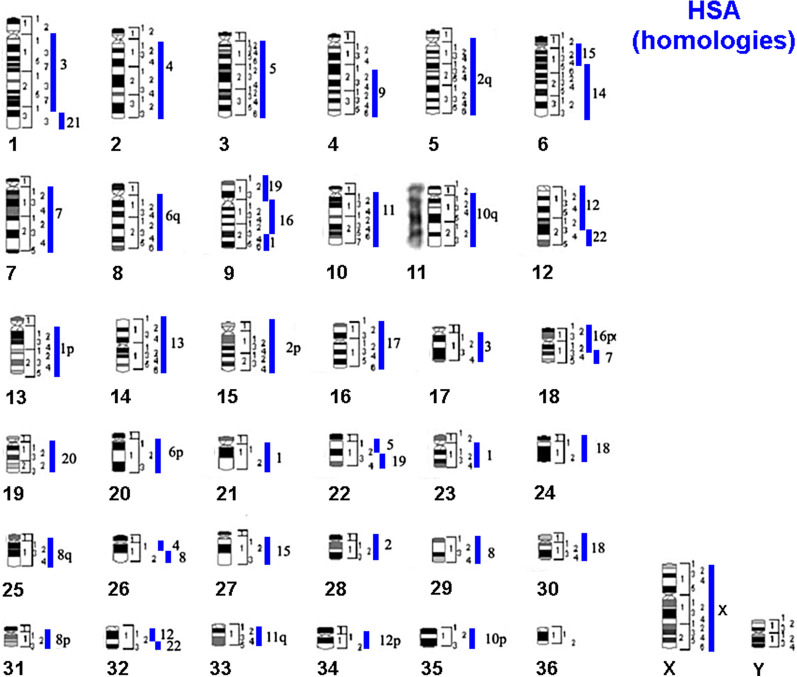
Idiograms of male dromedary showing regions of comparative homology with human chromosomes (HSA in blue) revealed by cross-species painting (acc. to [25]). Note that the positions of chromosomes CDR 4 and CDR 6 should be reversed according to the sorting data (see text)

In our previous study of cross-species painting in camelids [25] it was observed that the size of CDR 4 was greater than in other camelids, in that part of its long arm contained more GC rich heterochromatin than in related species. This is well illustrated in a camel:guanaco hybrid animal, which is heterozygous for this heteromorphic chromosome [25, 26]. These observations on the variable size of alpaca homologues were confirmed recently when we sorted the alpaca chromosomes and found that the VPA homologue of CDR 4 was in a lower position at VPA peak D in the alpaca flow karyotype than in the dromedary flow karyotype (Figs. 2 and 4). Chromosome painting between human and dromedary (Fig. 5) had previously provided a good comparative map [25], although the order of chromosomes CDR 4 and 6 in the published karyotype (reproduced here without change in Fig. 5) is mistaken, as CDR 6 should be CDR 4 and vice versa. These problems clearly suggested that an alternative strategy to painting was needed to make an accurate homology map between human and alpaca.

Our recent experience of using next generation sequencing (NGS) of sorted chromosomes to identify breakpoints in a t(9;14) translocation characteristic of the Ishikawa 3-H-12 tumor cell line [27] suggested a promising strategy. In this experiment, not only was a fusion gene demonstrated at the breakpoint, but it was possible also to identify individual homologues by the assignment of allelic variants in the sorted translocation. Separation by sorting of a heteromorphic chromosome 14 pair allowed the two homologues to be separately identified by sequencing.

**Fig. 6:**
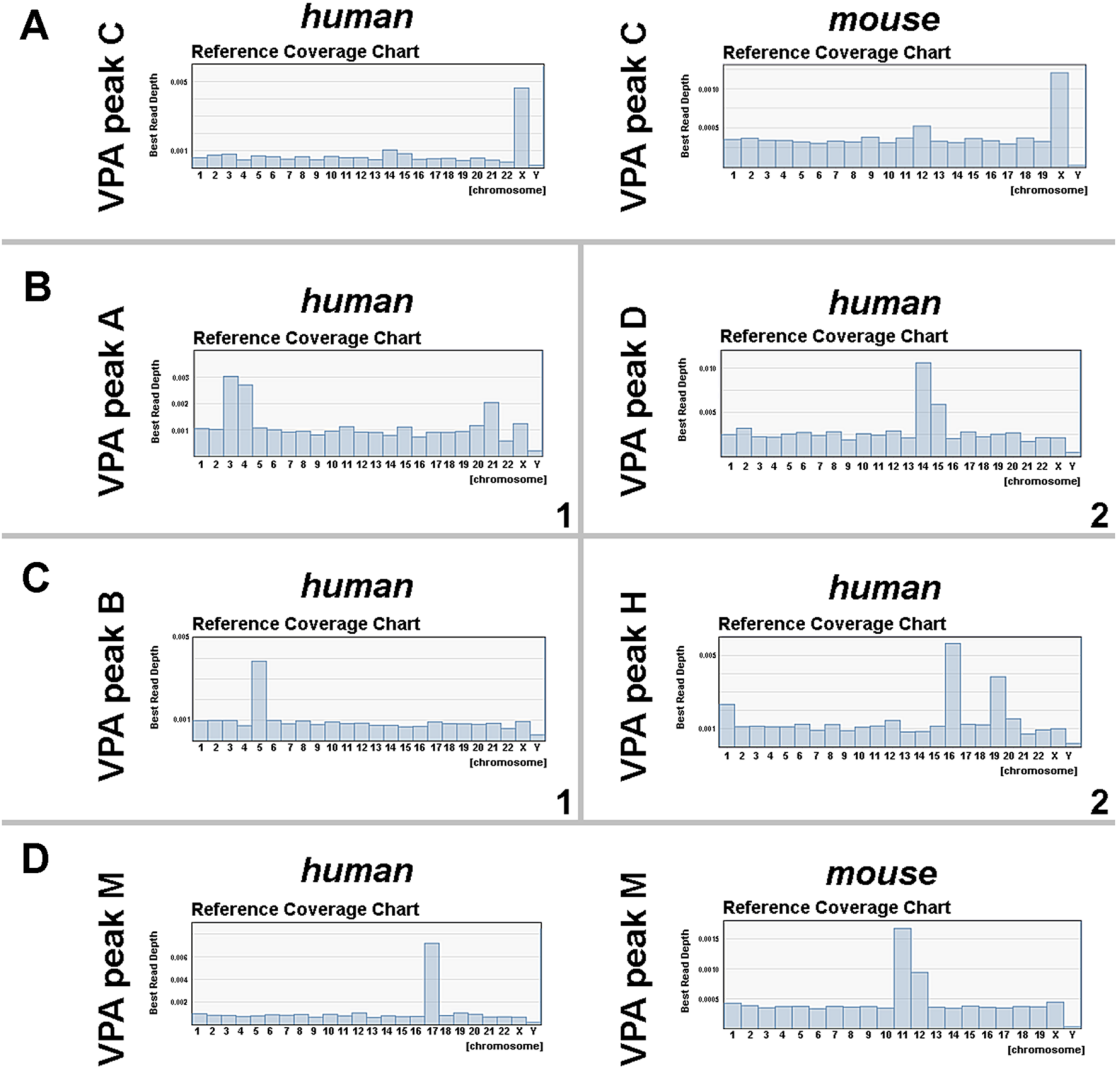
NGS reads from alpaca flow karyotype are shown; number/proportion of reads assigned to each human chromosome (HSA) are indicated in the X-axis. Non-specific reads are distributed equally between chromosomes. The results demonstrate strong chromosome homology between alpaca, mouse, and human based on chromosome-specific sequencing. (**A**) Alpaca X (= VPA peak C in flow karyotype) map to the reference human and mouse X chromosomes. (**B-1**). VPA peak A maps to human chromosomes 3, and 21 which shares homology with CDR 1, and to HSA 4 sharing homology with CDR 2. (These dromedary chromosomes are likely therefore to be conserved in VPA 1 and VPA 2, respectively). (**B-2**) VPA peak D shows homology to most of human chromosomes 14 and 15. HSA 14 and 15 are syntenic in most mammalian karyotypes, are both present in CDR 4 and are likely to correspond to VPA 6 (because CDR 4 has a large interstitial repeat, absent in alpaca, which is indicated by its larger size and its position in the flow karyotype above the CDR 3 peak, see text and Fig. 5). (**C-1**) VPA peak B seems to map only to human chromosome 5 which corresponds to CDR 3 and therefore to VPA 3. (**C-2**) VPA peak H maps to human chromosomes 1, 16 and 19 (all of which share homology with CDR 9, and therefore most likely to VPA 9 also). (**D**) VPA peak M maps to HSA 17 and MMU 11 and 12. As CDR 16 shares sequences with human 17 it is likely that it corresponds to VPA 16. MMU 12 has homology with parts of human chromosome 2, and 7 (probably present in VPA peak M) but not with HSA 17. This satisfactorily explains why the VPA peak M contains chromosome fragments corresponding to parts of both MMU 11 and 12 (see text and Fig. 7)

DNA amplification and DNase digestion of the sorted alpaca chromosomes generated small fragments of variable size, averaging about 300 base pairs. DNA sequencing of these fragments was carried out by the Ion PGM sequencer as detailed elsewhere [27] and a BLAST search was used to find similarity of sequence reads in the human genome reference (GRCh37/hg19) and mouse genome reference. The results in Figs. 6 and 8 show that sequenced alpaca DNA reads in convincing numbers can be aligned in the reference genome with specific human chromosomes. A small proportion of the sequences show equal amounts of non-specific matches across the whole human karyotype, irrespective of the origin of the sorted chromosome fragments and unrelated to the amounts of centromeric heterochromatin observed in the C-banded karyotype.

The alpaca X sequence reads map convincingly to the human and mouse Xs (Fig. 6A). It is interesting to note, that the syntenic blocks comprising parts of human chromosome pairs 3 and 21, and 14 and 15, are associated together in alpaca flow karyotype VPA peaks A and D, respectively (Fig. 6B); this is the case also in almost all tested mammals [6], except for human and mouse (*Mus musculus*, MMU, 2n = 20), in which alpaca VPA peak A has homology with MMU 3 and 16, and alpaca VPA peak D with MMU 2, 9 and 12. These regions are syntenic in the conserved chromosomes 1 and 4 of the dromedary (CDR 4 rather than 6, because of the large interstitial repeat noted above), indicating that they probably correspond also to chromosomes 1 (VPA peak A) and 6 (VPA peak D) in the alpaca (Fig. 5).

All the NGS reads from alpaca flow karyotype VPA peak B seem to map to human chromosome 5 (Fig. 6C) and this may be because VPA peak B matches CDR peak 3 in the dromedary. In the dromedary CDR 3 shares homology only with human chromosome 5 (*Homo sapiens*; HSA 5) (Fig. 5) and thus corresponds to VPA 3.

**Fig. 7:**
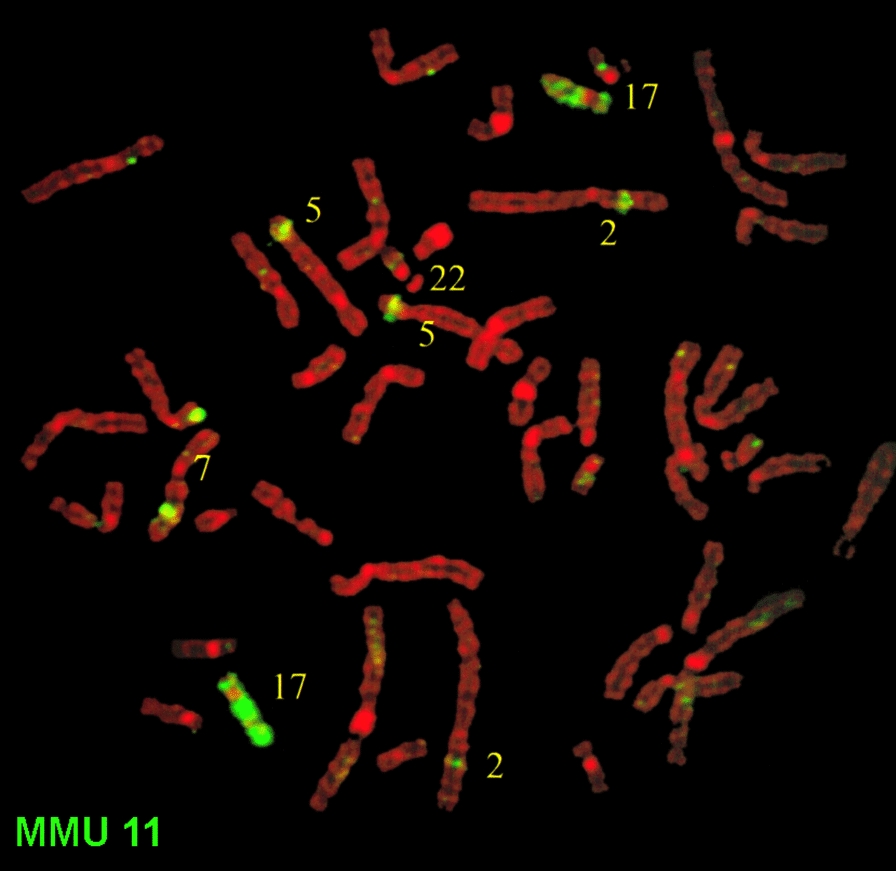
Human metaphase painted with mouse chromosome 11 probe (MMU 11) shows hybridization signals on human 17p & q, 5p14-15, 2p15-21, and 7q21-31, with weaker signals on 22q11 and some other chromosomes. No signals are observed on chromosome 12. Modified from [28]

Also of interest are the alpaca sequences from flow karyotype VPA peak M, which maps to most of HSA 17 and MMU 11 and 12 (Fig. 6D). HSA 17 and MMU 11, but not MMU 12, are known by genome sequencing to be homologous in these two species, and chromosome painting shows that mouse 11 probe MMU 11 produces strong hybridization signals on human 17p & q, 5p14-15, 2p15-21, and 7q21-31, with weaker signals on 22q11 [28] (Fig. 7). However, mouse 12 shares homology with different parts of HSA 2, and 7 (result not shown), and this suggests that alpaca VPA peak M contains these parts, perhaps incorporated in smaller chromosomes corresponding to CDR 18 and 28 (see Fig. 5). Thus, all the NGS matches are quite compatible with the earlier evidence of homology from chromosome painting and provide support to our view that chromosome-specific sequencing of chromosome-specific DNA fragments from sorted chromosomes is a reliable procedure for demonstrating cross-species homology.

**Fig. 8:**
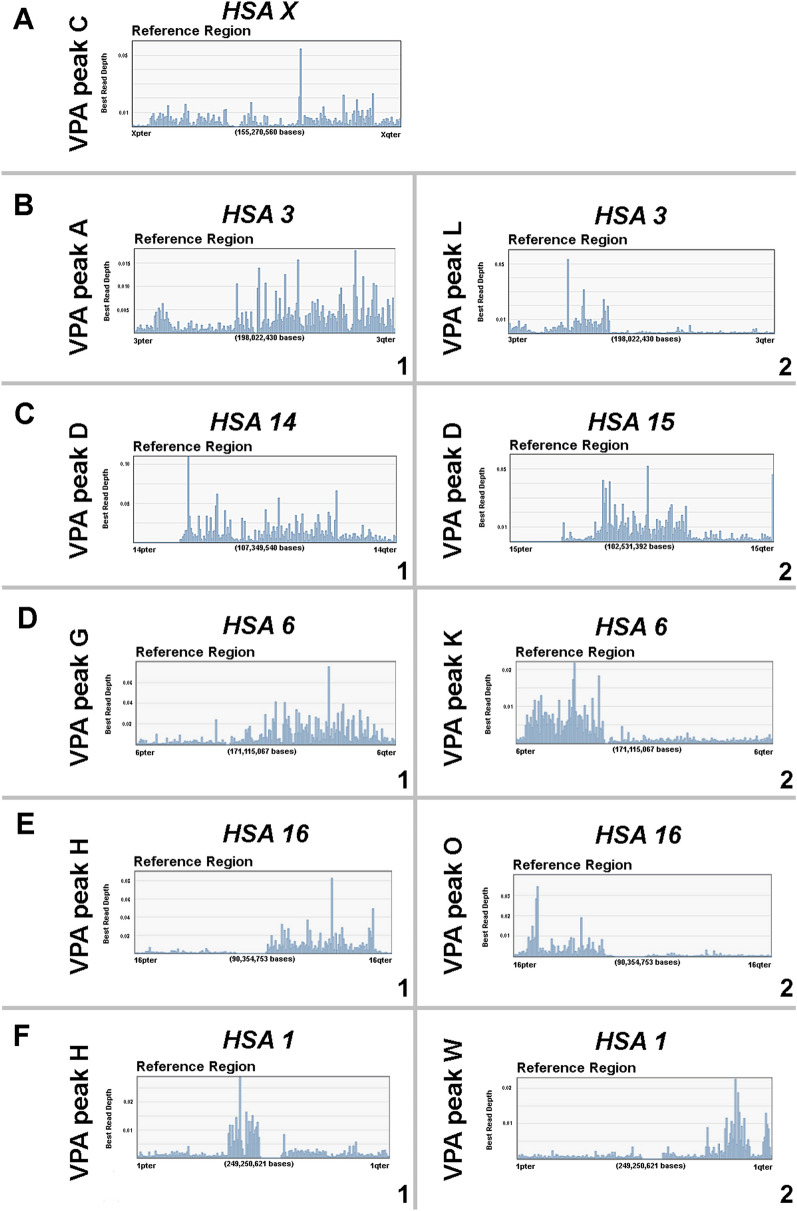
NGS reads from alpaca flow karyotype are shown as in Fig. 6—however, here chromosome-specific views are provided in reference coverage charts. (**A**) VGA peak C is distributed across the human X chromosome. Short to long arm is from left to right. The lack of signals at the distal short arm may correspond to a lack of homology with the human XY pseudo-autosomal (pairing) region. The gap about one third along to the right may be the site of the X centromere. (**B**) VPA peaks A (B-1) and L (B-2) aligned to reference human chromosome 3 show that the short arm of HSA 3 has homology to peak L and the long arm to VPA peak A, corresponding to CDR 1 and CDR 17 respectively. (**C**) VPA peak D show homology to most of human chromosomes 14 (C-1) and 15 (C-2) except for the short arms (left) of both human acrocentric chromosomes, which carry ribosomal and centromeric repeats. HSA 14 and 15 are syntenic in most mammalian karyotypes and are both present in CDR 4. (**D**) VPA peaks G (D-1) and K (D-2) are distributed across human chromosome 6 and map to CDR 18 and 20. The breakpoint appears distinct in HSA 6p but not in HSA 6q. (**E**) VPA peaks H (E-1) and O (E-2) are distributed across the long and short arms respectively of human chromosome 16. These regions share homology with CDR 9 and 18. The large gap between the two may in part be due to the large heterochromatic region of HSA proximal 16q. (**F**) VPA peaks H (F-1) and W (F-2) map to short regions of human chromosome 1 and share homology with short regions of CDR 9 and 21

The distribution of the chromosome-specific alpaca DNA fragments from NGS reads are shown along the length of each reference human chromosome in Fig. 8. In Fig. 8A the number of reads corresponding to the human X reference seem less than in the other distributions, although half are to be expected as only one X is present in the male alpaca cell line; in this case the position of the centromere may be identified as a narrow gap one third from Xpter. The lack of signals at the distal short arm may correspond to a lack of homology with the human XY pseudo-autosomal (pairing) region.

The centromeres and telomeres are evident in the reference coverage of the NGS reads, usually as regions with only a few matches (as these contain DNA repeats which are removed by an annealing step after DNA amplification of the sorted chromosomes and therefore are not fully represented in sequenced fragments). In Fig. 8C the low number of NGS reads from alpaca flow karyotype VPA peak D at the distal end (left) of the short arms of human chromosomes 14 and 15 are explained by the absence in the alpaca homologue of the repetitive ribosomal and satellite regions adjacent to the nucleolus organizers that are present in HSA 14 and 15. Similarly, no alpaca fragments map to the large species-specific regions of human chromosome 1q12, and 16q11.2, that are known to be heterochromatic (Fig. 8E and F). Where more than one alpaca chromosome peak shares homology with one human chromosome, the order and location of each region in the human chromosome is apparent and the gap between them is also indicated by fewer matches in the histogram or by the insertion of a syntenic block from another alpaca VPA peak (Fig. 8B, D and E). The distributions demonstrate the beginning and end of each syntenic block and point to the sequences where the breakpoint junctions might be found, as shown in Fig. 8B. The exact breakpoint may be determined with much greater precision than with chromosome painting by interrogating the chromosome-specific fragments on either side of the putative junction, as we have shown in our study of the Ishikawa 3-H-12 tumor cell line [27].

The results of our study on alpaca:human chromosome homology using sorted alpaca chromosomes are summarized in Table 1. This indicates that syntenic blocks on different pairs of human chromosomes show syntenic associations in alpaca chromosomes. The finding that the same syntenic associations are present by sequencing in alpaca and by chromosome painting in dromedary chromosomes is not only consistent with the high level of genome conservation among camelids, but also provides proof of principal that chromosome-specific sequencing is a reliable procedure for identifying cross-species homologies, particularly where karyotyping is problematic. Table 1 does not include results from all alpaca flow karyotype peaks; the missing peaks are mostly those containing the smaller alpaca chromosomes for which chromosome-specific DNA was not included in this preliminary study.

**Table 1:** Homologies of flow sorted VPA libraries/peaks and human (HSA), dromedary (CDR) and alpaca (VPA) chromosomes

VPA peaks	HSA	CDR	VPA (presumed)
A	3, 4, 21	1, 2	1, 2
B	5	3	3
C	X	X	X
D	14, 15	4	6
E	2, 14, 15	5, 4	5, 6
F	2, 7, 9, 17	5, 7, 4, 16	7, 4
G	6	8	8
H	1, 16, 19	9	9
J	13	17	17
K	2, 6	15, 20	15, 20
L	3	17	17
M	17	16	16
N	8	25, 26, 29, 31	16
O	16, 17	18	18
P	6, 16	18	20, 18
R	20	19	19
W	1	21, 23	21, 23

## Conclusion

It is accepted that chromosome homology maps can be constructed directly from the published whole genome sequence data, but these genomes are seldom complete and chromosome-specific sequencing has economic advantages and may play a valuable role in genome assembly. Meanwhile, it is hoped that the sequencing of flow sorted chromosomes, as presented here, will be as rewarding when applied to the construction of chromosome homology maps in more distantly related taxa, particularly across phyla, such as between monotremes and mammals, where data are seriously lacking.

## Data Availability

The datasets used and/or analysed during the current study are available from the corresponding author on reasonable request.

## Abbreviations

BLAST: Basic Local Alignment Search Tool
CDR: *Camelus dromedarius*, Dromedary
DNA: Deoxyribonucleic acid
HSA: *Homo sapiens*, Human
MMU: *Mus musculus*, Mouse
NGS: Next generation sequencing
VPA: *Vicugna pacos,* Alpaca
